# Recent Advances in β-Glucosidase Sequence and Structure Engineering: A Brief Review

**DOI:** 10.3390/molecules28134990

**Published:** 2023-06-25

**Authors:** Bei Ouyang, Guoping Wang, Nian Zhang, Jiali Zuo, Yunhong Huang, Xihua Zhao

**Affiliations:** 1 College of Life Science, Jiangxi Normal University, Nanchang 330022, China; 2 School of Computer and Information Engineering, Jiangxi Normal University, Nanchang 330022, China

**Keywords:** β-glucosidase, biocatalysis, biosynthesis, enzyme engineering

## Abstract

β-glucosidases (BGLs) play a crucial role in the degradation of lignocellulosic biomass as well as in industrial applications such as pharmaceuticals, foods, and flavors. However, the application of BGLs has been largely hindered by issues such as low enzyme activity, product inhibition, low stability, etc. Many approaches have been developed to engineer BGLs to improve these enzymatic characteristics to facilitate industrial production. In this article, we review the recent advances in BGL engineering in the field, including the efforts from our laboratory. We summarize and discuss the BGL engineering studies according to the targeted functions as well as the specific strategies used for BGL engineering.

## 1. Introduction

β-glucosidases (BGLs) are a class of enzymes that hydrolyze the β-1,4-glycosidic bond of the non-reducing terminal residue of β-d-glucoside while releasing glucose. BGLs have been applied in many biotechnological processes, such as the saccharification of lignocellulosic biomass for the production of bioethanol [1], wine and juice production to enhance flavor [2], and addition to feed to improve the digestion of cellulose in farm animals [3]. Additionally, BGLs play a role in cellulose digestion and phytohormone activation and participate in the hydrolysis of mammalian glucosyl ceramides [2].

**Figure 1:**
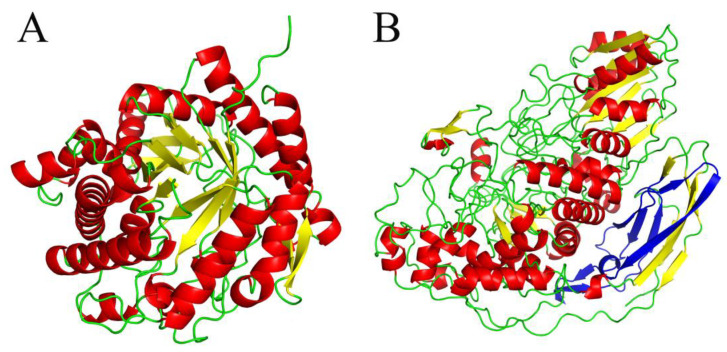
The three-dimensional structure of BGL. (**A**) Structure of PcBGL1A (PDB ID: 2e3z) from the GH1 *Phanerodontia chrysosporium* with α-helices, β-sheets, and loops shown in red, yellow, and green, respectively. (**B**) Structure model of 16BGL from the GH3 *Penicillium oxalicum* with α-helices, β-sheets, and loops shown in red, yellow, and green, respectively. The FnIII domain of unknown function is shown in blue.

There are two widely acknowledged classifications of BGLs at present: one based on substrate specificity and the other on BGL structural features. According to substrate specificity, BGLs are classified as aryl-BGLs (hydrolyzing only aryl-β-glucoside bonds), cellobiases (hydrolyzing only cellobiose), and BGLs with broad substrate specificity (hydrolyzing a wide range of substrates with different bonds, e.g., β(1→4), β(1→3), β(1→6), and α(1→6) bonds) [4]. In terms of BGL structural features, BGLs are mostly classified into two families: the glycoside hydrolase (GH) family 1, which presents a typical (α/β)_8_ TIM-barrel structure and pocket-like catalytic channel [5] (Figure 1A), and the GH family 3, which has a more complicated structure including the (β/α)_8_ TIM-barrel fold, the (α/β)_6_ sandwich domain, and the FnIII domain of unknown function [6] (Figure 1B). In general, BGLs from the GH1 family are widely found in archaea, plants, and animals, while GH3 BGLs are mostly from bacteria, fungi, and yeasts [4].

**Figure 2:**
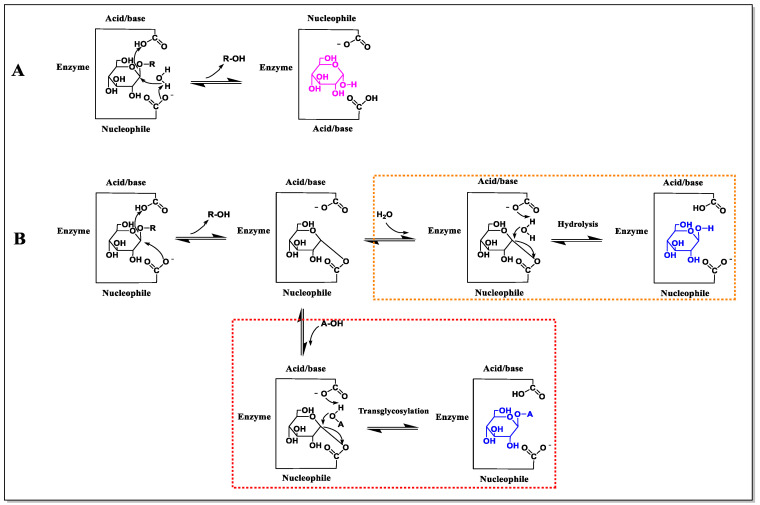
Reaction mechanism of BGL. (**A**) The “inversion” mechanism. (**B**) The “retention” mechanism. Both the leaving group (R) and the acceptor (A) are different glycosyl molecules.

BGLs catalyze hydrolytic reactions using either the “retention” or “inversion” mechanism (Figure 2). The inversion mechanism includes only a single step where the catalytic nucleophilic reagent of BGL deprotonates a water molecule (Figure 2A). This activated water molecule directly attacks the glycosidic bond to displace the aglycone and releases the sugar moiety with inversion of the configuration of the anomeric carbon [4].The retention mechanism consists of two steps: glycosylation and deglycosylation (Figure 2B). In the first step, the catalytic nucleophilic reagent attacks the allosteric carbon of the glucose residue as the glycosyl donor and forms the enzyme–glucose intermediate with the help of the catalytic acid that protonates the glycosidic oxygen [7]. The second step (orange box) involves a water molecule attacking the carbohydrate–enzyme linkage, transferring the proton to the active site acid/base carboxylate and releasing glucose.

In addition to breaking the glycosidic bonds in sugars, BGLs can also lead to the formation of glycosidic bonds (i.e., transglycosylation) in non-aqueous media with hydroxyl groups [8]. The reaction mechanism is similar to the BGL hydrolysis process, with a slight difference in the second step (Figure 2B, red box) which involves the attack of another sugar molecule (receptor A) rather than a water molecule, leading to transglycosylation [9]. The high added-value biomolecular products, such as alkyl-β- and aryl-β-glucosides as well as small-molecule oligosaccharides, have many promising applications in pharmaceuticals, chemicals, cosmetics, food, and detergents [10].

However, the rate of transglycosylation product synthesis by BGLs is extremely limited. Moreover, BGLs are most active only at 40–70 °C and pH 4.5–5, but industrial applications frequently go beyond these boundaries. For instance, the pre-treatment steps for removing lignin and other secondary wall components during biofuel production typically work at temperatures above 80 °C [11]. In flavor enhancement of fruit juice, enzymes with optimal activity at acidic pH 2.8–3.8 would be better adapted to release the glycosidically bound volatiles [7]. In the tandem cellulolysis process, the hydrolytic end product, glucose, inhibits BGL, leading to the accumulation of cellobiose, which in turn inhibits endo-1,4-β-d-glucanase (EG) and cellobiohydrolase (CBH). This phenomenon renders BGL the most critical enzyme for bioethanol production through biomass conversions [4].

To broaden the applications of BGLs in industry, it would be beneficial to enhance the capability of BGLs to tolerate non-mild conditions such as high temperatures, high concentrations of glucose, extreme pH, and high concentrations of organic solvents, to name a few [12,13,14,15]. In this paper, we review the recent advances in engineering BGLs with enhanced enzymatic properties. We outline the strategies used for BGL engineering as well as discuss the structural features and molecular mechanisms that lead to improved enzymes.

## 2. BGL Engineering Strategies

**Figure 3:**
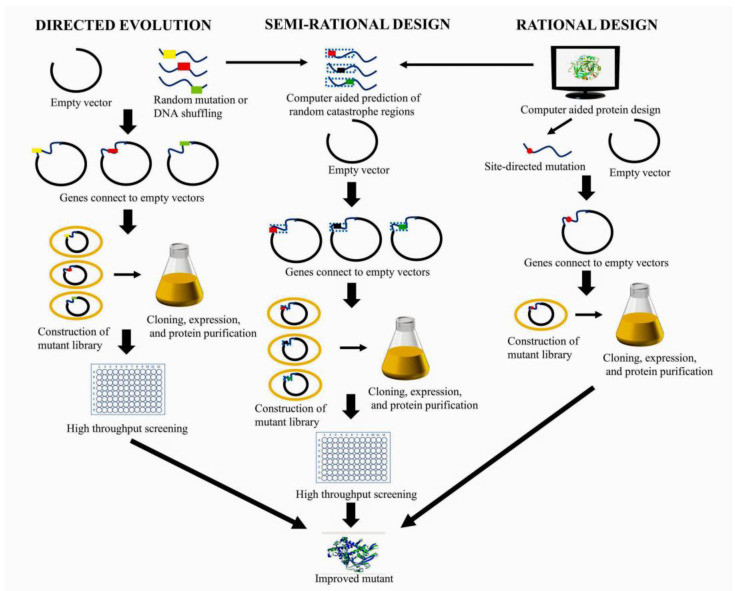
Directed evolution, computer-aided rational design and semi-rational design strategies for enzyme engineering.

Enzyme engineering strategies can be classified as nonrational, rational, or semi-rational, depending on the degree of the sequence, structure, and function information of a target enzyme that is taken into account (Figure 3). The most typical nonrational strategy is directed evolution [16,17], pioneered by Frances H. Arnold, laureate of the 2018 Nobel Prize in Chemistry. A common and effective strategy for rational or semi-rational design is computer-aided design, which can help reduce experimental costs and shorten development cycles [5]. All three strategies have been applied to engineer BGLs to improve their functionality (Table 1).

### 2.1. Directed Evolution

Directed evolution, an artificial procedure designed to mimic natural evolution, is an effective strategy for protein engineering in the absence of structural and functional knowledge. It accelerates the pace of mutagenesis, recombination, and protein selection in an explicit manner to obtain desired properties. The approach typically comprises an iterative cycle of mutagenesis to generate diverse mutants followed by high-throughput screening [37].

#### 2.1.1. Generation of Diverse Mutants

With advances in molecular biology tools and techniques, several mutagenesis strategies have been developed for DNA sequence diversification. Two natural evolutionary processes, random mutation and genetic recombination, have been employed to generate sequence diversification in vitro. Random mutagenesis is a non-recombination process in which one or more point mutations, additions, deletions, or inversions of the genome take place due to improper DNA replication or damage repair. The methods for generating sequence diversity of BGLs include error-prone PCR [38] and cassette mutagenesis [39]. Our laboratory has improved multiple enzymatic characteristics of 16BGL using error-prone PCR, including enhanced activity and product tolerance [20]. However, it is difficult for random mutagenesis to obtain highly active mutants. To address this, DNA shuffling—similar to the homologous recombination method—was introduced, which is a technique that includes Dnase I limited treatment of targeted DNA for primer-less PCR, PCR with primers, and construction of a mutant library. This technique was successfully utilized and increased the affinity of substrates for BGL [40]. Other recombination methods included the staggered extension process (StEP) [41], random-priming in vitro recombination (RPR) [42], and phage-assisted continuous evolution (PACE) [43], to name a few.

#### 2.1.2. Mutant Screening

Directed evolution necessitates a sensitive and efficient method for high-throughput screening of the huge number of mutants generated. Agar plate and 96-well microplate screening mainly account for traditional library screening. The agar plate-based screening exploits the direct correlation between host growth on selective agar plates and exocytosis of enzymes from cells into the solid medium with specific substrates for rapid screening of improved variants by the size of the halo [44]. The 96-well microplate is currently dominating the screening tests due to its flexibility in sample manipulation, low cost, and simple operation. A large number of protocols have been implemented by machine automation; however, in practice, the micro-titration plate method is limited to screening tens of thousands of clones [45].

In recent years, researchers have developed many efficient, precise, and sensitive methods for screening mutant libraries such as fluorescence-activated cell sorting (FACS), microfluidics technology, and deep mutation scanning (DMS). FACS, an advanced high-throughput screening technique, allows rapid screening and sorting of cells or other suspension particles according to their fluorescent features [46]. Hardiman et al. used FACS to screen BGL mutants with higher specificity and catalytic efficiency [47]. However, due to its high non-specific background, FACS necessitates further rescreening and can only be used to analyze intracellular or membrane-bound products that produce a fluorescent signal associated with target compounds. The analysis of extracellular products is challenging when using FACS [48]. All these advanced methods require complex setups and sufficient expertise in microfluidics, optics, electronics, and programming to fully operate the functional sorting equipment. Recently, Yao et al. reported a red-emission probe for the determination of BGL synthesized through conjugating a glucoside to an aggregation-induced emission (AIE) fluorophore, which offers intriguing ideas about screening for highly active BGLs [49].

#### 2.1.3. Machine Learning-Assisted Directed Evolution

Most of the time, directed evolution is limited by the fact that even the most high-throughput screening or selection methods sample only a small portion of the sequence space for hits with improved function and discard unimproved sequences. It has been shown that machine learning (ML)-assisted directed evolution methods can use the information extracted from these unimproved mutants to accelerate evolution and expand the number of enzymes that can be optimized for desired performance [50]. In addition, ML has been used to more intelligently navigate sequence space during directed evolution of protein function, and to produce proteins from scratch that satisfy sets of constraints associated with binding interfaces [51]. Computational power is consistently increased, while the sequencing costs and time continue to decline. Therefore, ML-based targeted evolutionary applications will become more feasible and fuel the engineering of BGL enzymes.

### 2.2. Rational Design

Though directed evolution is powerful, it is still challenging to obtain improved mutants because of the complexity of building a sensitive and efficient screening system as well as the high workload of screening huge mutant libraries. Computer-aided rational design has become a favored strategy. It entails a thorough comprehension of active sites and functions of enzymes, and specific residues are selected for targeted mutagenesis. The three main methods used to help identify mutation-specific residues are structural analysis, multiple sequence alignment (MSA), and robust computational techniques [37].

#### 2.2.1. Structural Analysis

In most cases, molecular modeling of proteins is the first step in structural analysis. Currently, a significant number of protein structures are being registered in the Protein Data Bank (PDBs) and the AlphaFold Protein Structure Database [52] to facilitate homology modeling studies. The sequence similarity of newly discovered proteins can model structures based on sequence comparisons with similar protein sequences as long as the sequence similarity is high enough (>25%). There are several famous homology modeling tools available, such as Yang Zhang Lab (https://zhanggroup.org/, accessed on 1 May 2023), Swiss-Model [53], or Rosetta [54].

Subsequently, structural analysis methods can select mutation sites by comparing structures with high/low protein sequence homology or by observing the structures surrounding active pockets. In general, observation of the structures around active pockets can be achieved by docking the substrate to the enzyme. Commonly used docking tools are AutoDock [55], AutoDock Vina [56], Glide [57], and GOLD [58].

#### 2.2.2. Multiple Sequence Alignment (MSA)

For desired modifications, conserved amino acid residues are identified by alignment with other related sequences. Residues with low conserved scores, which are not essential for enzymatic structure and function, can be utilized as targeted sites for modification. Common MSA tools include ClustalW, Clustal Omega, MAFFT, T-Coffee, etc. [59]. Since proteins’ structures evolve more slowly than their sequences, integrating structural information in MSA can enhance the quality of alignment and provide more reliable data for analysis. Structure-based MSA tools include 3D-COFFEE, EXPRESSO and MICAlign [60].

#### 2.2.3. Computational Approaches

With advances in computational biology, many strategies for identifying protein engineering hotspots have been developed. Molecular dynamics (MD) simulations, which help predict mutation sites by providing atomic information about dynamic molecular interactions that determine protein properties, have achieved significant breakthroughs with the use of graphical processing units over the years [61]. Virtual screening is used to find potential candidates through a fast search of large enzyme libraries based on computational simulations, which is considered a promising alternative computational design approach before the experimental screening of mutant libraries [62]. The neural network-based model AlphaFold2 [63] provides help in identifying amino acid substitution sites by accurately predicting protein structures, while Rosetta, developed by Baker, also provides assistance by modeling protein–protein complexes, docking small molecule ligands into proteins [64].

However, it is impossible for MD simulations or Rosetta to typically capture the data about the overall global behavior and properties of proteins [65]. A data-driven approach of ML combined with statistics overcame this disadvantage by inferring the numerous and possibly unknown factors which map from sequence to function according to the above data and provided superior predictive accuracy for predicting mutation sites in BGL [66]. For example, a regression model based on a dual-input convolutional neural network was used to predict the binding affinity of cellulase to the substrate to improve enzyme activity [67]. Due to the lack of negative sequence examples in the DMS dataset, and the inability to learn directly from the large-scale sequence function DMS dataset with the ML-supervised method, a method was developed to classify the DMS dataset as positive unlabeled data and successfully applied to design thermally stable BGLs [68]. It is believed that the availability of computational tools such as ML will become more crucial along with screening capacity, computational power, and increased workload.

#### 2.2.4. Site-Directed Mutagenesis (SDM)

SDM is an important tool in protein engineering and is also known as oligonucleotide-specific mutagenesis or site-specific mutagenesis. Typical SDM methods are the overlapping extension PCR (OEP) and the whole-plasmid single-round PCR. OEP uses complementary primers to amplify two DNA fragments with overlapping ends [69]. Sun et al. used OEP to obtain a BGL mutant with higher glucose tolerance than the wild type [13]. However, the long-length PCR products, which are used as primers, limit the amplification efficiency in OEP. Guo et al. introduced an improved OEP method to increase the amplification efficiency of long-length multisite directed fragments by providing primers continuously [70]. The whole-plasmid single-round PCR method is involved in PCR replication of double-stranded plasmid DNA template using two complementary primers with the desired mutation for better glucose tolerance [37].

### 2.3. Semi-Rational Design

Semi-rational design exploits the advantages of directed evolution and rational design and requires information about protein sequence, structure and function with predictive algorithms. Several targeted sites are identified and mutated to form “smart” libraries and to obtain the desired mutants [71]. Therefore, the mutant libraries generated by this approach are usually small. Semi-rational design is generally achieved by directed evolution and site-saturated mutagenesis. Two web-based computational tools are worth mentioning, the HotSpot Wizard server which combines information from extensive sequence and structure database searches with functional data to create a map of mutation candidate residues for enzymatic activity of BGL [72,73], and Consensus Finder which uses a consensus sequence approach to identify the most frequently occurring amino acids, to replace rarely occurring amino acids, and to achieve BGL with high thermostability [36,74]. In addition to sequence-based design strategies, there exists effective methods based on protein structural information, such as combinatorial active-site saturation test [75], and focused rational iterative site-specific mutagenesis [76]. Both methods involve saturation mutagenesis at sites lining the binding pocket with the help of in-silico methods such as ML [77]. Similarly, both MD simulations and ML are valuable tools for efficiently exploring the effects of amino acid substitutions on protein structure and function in semi-rational design.

## 3. Engineering of BGL Functionalities

### 3.1. Enhancing Activity

Table 2 summarizes examples of BGL activity enhancement in recent years. Currently, computer-assisted semi-rational design is considered a very promising strategy for improving BGLs activity based on its advantage of balancing the size of the mutant library to reduce screening effort while obtaining the desirable results [73]. In addition, the molecular mechanisms of BGL activity enhancement have been revealed in order to engineer BGL [73,78]. Some studies focus on engineering the active-site tunneling residues. The BGL–cellobiose complex is mainly stabilized by hydrogen-bonding and hydrophobic interactions between cellobiose and side chains of amino acids located at the glycone (−1) and aglycone (+1) sites, such as the conserved Tyr320 from Neosartorya fischeri (NfBGL) [34]. BGL activity is also shown to be related to high glycosylation, as deglycosylation results in a significant decrease in enzymatic activity [10]. BGL has also been studied to focus on the loops and residues interacting with substrates. Higher substrate affinity with BGL may be due to the F256 binding residue that is located on a shorter loop [79]. The Exiguobacterium marinum BGL was found to be able to process longer cello-oligosaccharides, which is atypical in the GH1 family, and the discovery of this structure at the topologically specific catalytic interface provides a theoretical basis for designing BGLs with a strong capacity for cleaving cellulose oligosaccharides [80].

### 3.2. Improving Product Tolerance

The tandem enzymatic saccharification of lignocellulose releases the product (glucose) that inhibits BGLs, leading to the accumulation of cellobiose which further inhibits CBH and EG. Therefore, excellent BGL enzymes should be able to tolerate high-concentrations of glucose. Here, we summarize some mechanisms and strategies of the glucose-tolerant and stimulated BGL action discovered in recent years.

The majority of glucose-tolerant BGLs belong to the GH1 family, because most GH1 BGLs have a narrow and deep substrate binding pocket which is difficult for glucose to enter, and this binding pocket can be binned into three regions: glycone-binding site (−1 subsite), aglycone-binding site (+1 and +2 subsites), and the gatekeeper region [84]. Based on this property, the molecular mechanism of BGL product tolerance suggests that increasing the hydrophobicity of the aglycone-binding sites (+1 and +2 subsites) in the active site tunnel and the hydrophobicity and steric properties of the non-conserved residues in the gatekeeper region can improve BGL product tolerance. Other inactive sites are also associated with high tolerance of BGL products, including separate glucose binding sites [85] and some active channel residues [86]. Table 3 summarizes examples for enhancing product tolerance of BGLs in recent years.

A low concentration of glucose stimulates BGL activity, which may be caused by alleviating substrate inhibition [88]. A novel mechanism of BGL stimulation by “saccharide capture” through the response of the monosaccharide secondary binding region to alter the size of the catalytic gap entrance has also been recently proposed [93]. Indeed, the stimulation of BGL activity in response to glucose is sometimes not clearly explained by a single mechanism. The stimulation phenomenon may be explained by a competitive mechanism of glucose with the nonproductive binding of substrate and by transglycosylation, and the possible contribution of the two mechanisms in the activation by inhibitor was dependent on the rate-limiting step of glycosidic bond hydrolysis as well as on whether and which glucose-unit-binding subsites are interacting [94].

In brief, studies on glucose tolerance and stimulation of BGL have attributed this phenomenon to several mechanisms, including metastable effects, transglycosylation, disinhibition of substrates, and unbinding of nonproductive substrates. In addition, a relatively complete database of glucose tolerance of BGL products, Glutantβase, provides valid information for its rational design [91].

### 3.3. Improving Transglycosylation

In nature, glycosylation is mainly accomplished by Leloir glycosyltransferases [95]. However, glycosyltransferases are not ideal enzymes for glycosylation reactions due to their costly and complex processing. Glycosidic bonds also come to be formed through kinetically controlled transglycosylation of BGLs (Figure 2) with broad specificity and high stereoselectivity for natural or engineered substrates [96]. To improve the transglycosylation-to-hydrolysis (T/H) ratio, an effective strategy is to restrict the hydrolysis reaction to transglycosylation. As shown in Table 4, the rational design of targeted BGL catalytic tunneling of subsite residues provides methodological strategies: (1) reducing the binding in glycone (−) subsites; (2) increasing the affinity in aglycone (+) subsites; and (3) disrupting the binding of catalytic water: mainly by removing the hydrogen-bonding interactions with the catalytic water and the retention of nucleophilic water molecules at key amino acid residues, or enhancing the hydrophobicity at the active site entry or acceptor subsite [95]. For example, the Hydropathy Index For Enzyme Activity (HIFEA) strategy to reduce the hydrophilic index of BGL amino acid residues has been used for the rational design of oligosaccharide synthesis [28].

### 3.4. Improving Thermostability

BGL plays a key role in consolidated bioprocessing; however, high temperature causes BGL denaturation [11,99]. A comparative analysis on the enzymatic properties and amino acid composition of mesophilic, thermophilic, and hyperthermophilic BGLs revealed a number of factors that contribute to the thermal stability of proteins, such as hydrophobic effects [100,101], hydrogen-bonding and electrostatic interactions [100,102], aromatic interactions [100,103], protein structural densification [100], reduction in unfolding entropy [99,101], etc.

Structural comparison revealed that thermophilic and hyperthermophilic enzymes are more rigid than mesophilic enzymes. Since high temperatures unfold the highly flexible regions of the proteins quickly, flexibility can be used as an indicator to identify potential areas for modification to improve the thermal stability of BGLs [104]. Most of the current studies target mutations in flexible regions to increase protein rigidity and thermal stability. Common experimental methods used to determine protein flexibility include high-resolution ultrasound spectroscopy, pressure perturbation calorimetry, and nuclear magnetic resonance spectroscopy [100]. With the improvement of algorithm optimization and computational capacity, a series of bioinformatics software has been developed for analyzing protein structures and predicting flexible regions, as shown in Table 5.

After the flexible regions are identified, two main strategies are used to rigidize the flexible region to improve thermostability. One strategy is to enhance the stability of the enzyme by reducing the conformational entropy in the unfolded state [100], and common methods include introducing disulfide bonds in the flexible region or replacing glycine with the most rigid proline. It has been proposed that truncating the flexible loops of the protein can also reduce the conformational entropy of BGL [104]. Another strategy is to stabilize the folded state by increasing favorable interactions (including hydrophobic interactions and hydrogen bonds, etc.) or removing negative interactions. There are also studies focused on increasing the densification of the BGL structure (by truncating the carbohydrate-binding domain) [111].

The most crucial thing is to assess the thermostability of mutants at high temperatures. A common strategy is to perform melting temperature (T_m_) analysis by differential scanning fluorescence or differential scanning calorimetry. In addition, the unfolded Gibbs free energy (ΔG) can also measure stability. T_m_ and ΔG can be predicted by computational tools based on force fields and ML, such as FoldX, Rosetta Design, etc. [107]. However, studies also stress the need for new computational tools that can not only more accurately predict significant changes in thermal stability, but also extend to more refined moderate changes [112].

In brief, the factors affecting enzyme thermal stability suggest a series of methods and strategies for identifying flexible regions and protein rigidity, as well as computational methods for assessing thermal stability properties of mutants. Table 6 summarizes the instances on BGL thermal stability engineering in recent years. We hope this summary will benefit the development of computational predictors for engineering the thermostability of enzymes including BGLs.

### 3.5. Improving Catalytic Performance in Unconventional Phase

Maintaining high activity in unconventional phase (e.g., ionic liquid (IL), saline concentrated seawater, or organic solvents) is critical for applications of BGLs in lignocellulosic biocatalysis, saline land improvement, and marine cellulose biomass utilization [15,117]. Therefore, it is important to understand the mechanisms that BGLs can tolerate in the unconventional environment during catalysis and use these mechanisms to guide the discovery of BGLs with higher activity/stability.

Surface charge engineering is a promising approach from the perspective of IL tolerance. Mutants with increased negative surface charge showed higher catalytic efficiency in IL due to the electrostatic repulsion between IL and the salt-bridge network of BGL [15]. The salt tolerance of mutants is improved by increasing the acidic amino acids on the protein surface and near the entrance of the active site to hinder the entry of high concentrations of salt ions into the active site [13,78]. Additionally, the increased electrostatic interactions may be responsible for organic solvent tolerance [118]. In addition, semi-rational design would be a promising approach to further explore the molecular basis of the BGL activity and stability in unconventional phase catalysis.

### 3.6. Improving pH Stability

The pH stability of BGLs is crucial for industrial applications including brewing, feed making, and paper making [119]. However, most natural BGLs are limited to a relatively narrow pH range (4.5–5) with optimal activity. It is necessary to engineer BGLs with altered or broadened pH performance. The optimal pH value of BGLs is widely attributed to the pK_a_ of catalytic residues. Although the PROPKA software makes it simple to estimate the pKa’s of ionizable residues [120], the estimation may not be sufficiently accurate for practical use because pK_a_ is regulated by a few complex factors and the active-site microenvironment. Therefore, it is still challenging to alter the pH performance of an enzyme by merely changing the pK_a_ value of the catalytic residue [14]. The high pH tolerance of BGLs may be related to the presence of a large number of surface-accessible negatively charged residues that keep the protein hydrated and protect the protein core from hydroxyl ion attack [114]. SDM of charged amino acids near the catalytic residues has broadened the pH activity distribution of BGLs [121]. With more detailed molecular mechanisms uncovered, BGL mutants with better pH properties can be designed in the future.

## 4. Conclusions

In conclusion, we provide a brief review of recent advances in approaches and functionality studies for engineering BGLs with better properties for biotechnological and industrial applications. Directed evolution continues to be a valuable solution for BGL engineering, but it is of note that a sensitive and efficient method must be developed for high-throughput screening of large mutant libraries. Advances in screening strategies will further strengthen the application of directed evolution for BGL optimization. With the rapid advancement of structural and computational biology, the experimental structures as well as the high-quality structural models (e.g., AlphaFold2 models) for BGL enzymes are now available, making computer-aided rational design a more preferred choice for BGL engineering by biochemists. With more and more functional assay data accumulating, it is also desirable to develop data-driven approaches such as ML algorithms to fuel BGL engineering.

Indeed, more and more targeted glycoproteomic studies are emerging to gain insight into the effects of glycosylation modifications on the enzymatic properties of BGL. This has not only provided a novel perspective on efforts to improve the activity and stability of BGL but has also inspired and encouraged other researchers to explore the field of glycoengineering of BGL. In addition, with the accumulation of more and more protein data and the huge screening workload, it is believed that artificial intelligence tools such as deep learning that can leverage more big data will be the mainstay to power the engineering of BGLs and even open up the possibility of de novo design of BGL in the future.

## Figures and Tables

**Table 1:** Summary of BGL engineering approaches.

Organism Source	Engineering Methods	High Throughput Screening Method	Improved Characteristics	Reference
*Clostridium thermocellum* (BglA)	Directed evolution Error-prone PCR	Assay medium screening method (0.02% Magenta GlcA)	Thermostability: ↑ T_i_ by 6.4 °C.	[18]
*Alteromonas* sp. L82 (*bgla*)	Rational design Site-directed mutation	---	Glucose tolerance: 40.7% of relative activity (glucose: 4 M).	[13]
Metagenomic library of Turpan Depression (*bgl1317*)	Rational design Site-directed mutation	---	Activity: ↑ by 80%. Glucose tolerance: *IC*_50_ from 0.8 to 1.5 M.	[19]
*P. oxalicum 16* (16BGL)	Directed evolution Error-prone PCR	Assay medium screening method 6-(β-d-glucopyranosyloxy)-7-hydroxy-2H -1-benzopyran-2-one	Activity: ↑ specific activity 70% at 40 °C.	[20]
*Thermotoga naphthophila* RKU-10 (TN0602)	Rational design Site-directed mutation	---	Transglycosylation: ↑ GOS productivity by 50%.	[9]
Soil Macrogenome Library (Bgl15)	Directed evolution Error-prone PCR	Double assay medium screening method (0.1% hesperidin)	Glucose tolerance: *IC*_50_ from 0.04 to 2.1 M. Thermostability: T_1/2_ from 0.8 h to 180 h at 50 °C.	[21]
*Trichoderma harzianum* (ThBgl)	Rational design Site-directed mutation	---	Glucose tolerance: 300% of relative activity (glucose: 0.25 M). pH: stability at broad range (pH 4–9).	[22]
*Caldicellulosirutor* *Saccharolyticus* (CsBglA)	Semi-rational design Site-directed mutation	Cell surface display Fluorescence detection medium screening method (pNPG)	Activity: ↑ K_cat_/K_M_ by 150% at 55 °C.	[23]
GenBank FJ686869 (Bgl1D)	Directed evolution Error-prone PCR	Assay medium screening method (0.1% hesperidin)	Activity: ↑ K_cat_/K_M_~23-fold. Thermostability: ↑ T_1/2_~10-fold.	[24]
*Paenibacillus* *polymyxa* (Glu1C)	Rational design Site-directed mutation	---	Thermostability: ↑ ~4-fold. Glucose tolerance: from 50% to 75% active at 1 M glucose.	[25]
*Talaromyces* *amestolkiae* (BGL-1)	Rational design Site-directed mutation	---	Transglycosylation: ↑ epigallocatechin gallate productivity by 48.8%.	[26]
*Trichoderma reesei*	Directed evolution (UV light, N-methyl-N′-nitro-N-nitrosoguanidine)	Detection medium screening method (phosphoric acid-swollen cellulose)	Activity: ↑ ~5-fold.	[27]
*T. reesei* (*Tr*Cel1b)	Rational design (Hydropathy index for enzyme activity) Site-directed mutation	---	Transglycosylation: ↑disaccharides productivity by 3.5-fold.	[28]
*Bacillus* sp. D1 (BglD1)	Semi-rational design Site-directed mutagenesis	---	Transglycosylation: ↑ GOS productivity by 11.5%.	[29]
Marine microbial metagenomic library (Bgl1A)	Semi-rational design Site-directed mutagenesis	---	Glucose tolerance: ↑ *IC*_50_~1.4- to 2.4-fold. Thermostability: ↑ T_1/2_~4.3-fold.	[30]
*Lentinula edodes* (LXYL-P1)	Rational design Site-directed mutagenesis	---	Activity: ↑ ~3-fold.	[31]
*Penicillium piceum* H16	Rational design Site-directed mutation	---	Thermostability: ↑ by 46.3%.	[32]
*Thermotoga* *Neapolitana* (*Tn*Bgl1A)	Rational design Site-directed mutation	---	Transglycosylation: ↑ by 7-fold.	[33]
*Neosartorya* *fischeri* (NfBGL)	Rational design Site-directed mutation	---	Activity: ↑ by 8%.	[34]
A dairy run-off metagenome (BG3L)	Rational design Site-directed mutation	---	Activity: ↑ ~2 or 3-fold.	[35]
Metagenomic library of Turpan Depression (Bgl6-M3)	Semi-rational design Site-directed mutation	---	Thermostability: ↑ T_1/2_~20-fold. Activity: ↑K_cat_/K_M_~5.6-fold. Glucose tolerance: ↑ ΔIC_50_ of 200 mM.	[36]

**Table 2:** Summary of protein engineering studies to improve BGL activity.

Organism	Strategy	Mutations	Molecular Effects	Improved Characteristics	References
*Halothermothrix orenii* (B8CYA8)	Rational design OEP	V169C, I246A	Lack of stable polar contacts; Reduction in side chain volume	Specific activity ↑ ~2-fold.	[81]
*Coniophora puteana* (CpBgl)	Semi-rational design (HotSpot, Alanine scanning technique) SDM	Q20C, A240S	A combination of structural changes in the active pocket and protein–ligand interactions	↑ By 65.75% and 58.58%, respectively.	[73]
*Chaetomella raphigera* (D2-BGL)	Directed evolution Error-prone PCR	F256M/Y260D /D224G	F256 and Y260 on a short loop related to the high substrate affinity of the enzyme	↑ ~2.7-fold.	[82]
Metagenomic library of Turpan soil (*Bgl1317*)	Rational design SDM	A397R, L188A, A262S	Increase in the polarity of residues and hydrogen bonding contacts	↑ By 80%.	[19]
*Talaromyces leycettanus* JCM12802	Rational design OEP	M36E, M36N, F66Y, E168Q	Increase in hydrophobic stacking interactions and hydrogen bonding networks of active centers	↑ ~1.4–2.3-fold.	[83]
*P. oxalicum 16* (16BGL)	Directed evolution Error-prone PCR	M280T/V484L /D589E	Increase in the number of hydrogen bonds formed by the substrate to increase the binding free energy	↑ By 22%.	[6]
*C. saccharolyticus*	Directed evolution Error-prone PCR, Random drift mutagenesis	---	Smaller residues near catalytic residues allow more flexibility in the active site or more access to the substrate	↑ ~2-fold.	[47]
*P. oxalicum 16* (16BGL)	Directed evolution Error-prone PCR	G414S/D421V/ T441S	Tighter active site pockets	↑ Specific activity 70% at 40 °C.	[20]
*Pyrococcus furiosus* (CelB)	Directed evolution DNA shuffling	N415S	---	↑ Up to 3-fold.	[40]
*C. saccharolyticus* (CsBglA)	Semi-rational design (SDM combined with random mutagenesis)	L64R/Y73F/ T221N/H324L	---	↑ K_cat_/K_M_ by 150% at 55 °C.	[23]

**Table 3:** Summary of protein engineering studies for enhancing product tolerance of BGLs.

Organism	Strategy	Mutations	Molecular Effects	Improved Characteristics	References
Metagenomic library of Turpan soil (*Bgl1317*)	Rational design SDM	L188A, A262S	Active site metastable interactions	*IC*_50_ from 0.8 to 1.5 M.	[19]
*Agrobacterium* *tumefaciens 5A* (H0HC94)	Rational design SDM OEP	W127F, C174V, V176A, L178A, L178E, H229S	Increase in the hydrophobicity of the aglycone-binding sites and gatekeeper regions	↑ ~2.2-fold	[87]
*Trichoderma* *Harzianum* (ThBgl)	Rational design SDM	L167W/P172L	Replacement of gatekeeper residues to alter active site accessibility	300% of relative activity (glucose: 0.25 M).	[22]
*T. Cel1A* (Bgl II)	Rational design SDM OEP	L167W/P172L	Replacement of gatekeeper residues to narrow the entrance to the active pocket	*IC*_50_ = 650 mM.	[88]
*Humicola insolens* (Bglhi)	Directed evolution Error-prone PCR	H307Y, D237V, N235S	Increasing trans-glycosylation Unbinding of unproductive substrates	---	[89]
*A. tumefaciens 5A*	---	---	Presence of separate glucose binding sites	---	[90]
Marine microbial Metagenome (SrBGL)	Rational design SDM	H228T	Interaction leading to glucose excretion by slingshot mechanism	↑ Affinity score for cellobiose.	[91]
*H. orenii* (B8CYA8)	Rational design SDM	V169C/E173L/ I246A	Increasing backbone kinetics of active channel residues and flexibility of active site pockets	75% of specific activity in 1.0 M glucose.	[84]
GenBank MK490918 (Bgl15)	Directed evolution Error-prone PCR Petri-dish-based double-layer high-throughput screening	S167V/W178L	Increasing transglycosylation activity	*IC*_50_ from 0.04 to 2.1 M.	[21]
Marine bacteria (*bgla*)	Rational design OEP	F171W	Increase in volume of side chains near the active site	40.7% of relative activity (glucose: 4 M).	[13]
Hot-spring metagenome (BglM)	---	---	The narrow space between the remnants of the gatekeeper’s base	---	[92]

**Table 4:** Protein engineering to enhance BGL transglycosylation.

Organism	Strategy	Mutations	Molecular Effects	Improved Characteristics	References
*T. amestolkiae* (BGL-1)	Rational design SDM	E521G	Stimulating glycosyl donor departure. Absence of side chains to reduce steric hindrance	↑ Epigallocatechin gallate productivity by 48.8%.	[26]
*T. naphthophila* RKU-10 (Tn0602)	Rational design SDM	F226G/F414S	Reducing steric hindrance and removing interactions at the aglycone-binding sites	↑GOS productivity ~1.3-fold.	[97]
*T. naphthophila* RKU-10 (Tn0602)	Rational design SDM	F414S	Improving hydrophilicity of the lumen of the −1 subsite	↑GOS productivity by 50%.	[9]
*Thermotoga maritima* (TmBglA)	Rational design SDM	N222F/Y295F /F414S	Creating a more suitable environment for hexanol in the active center pocket to inhibit hydrolysis	Hexyl-β-glycoside productivity from 14.49 to 22.8 mM.	[96]
*A. niger* (BGL1)	Directed evolution Error-prone PCR	Y305C	Reducing hydrolytic activity	K_i_ from 2.98 to 4.78 mM.	[98]
*T. neapolitana* (*Tn*Bgl1A)	Rational design SDM	N220F, N220R, N220Y	Inhibiting hydrolysis	Transglycosylation/hydrolysis from 0.33 to 1.45–2.71.	[95]
*T. reesei* (*Tr*Cel1b)	Rational design SDM HIFEA Strategy	I177S/I174S/ W173H	Inhibition of hydrophilicity of key amino acid residues in the catalytic sites	↑ Disaccharides productivity by 3.5-fold.	[28]

**Table 5:** Methods for improving protein stability.

Method	Access	Description	Reference
Constraint network analysis (CAN)	---	Local and global flexibility/stiffness properties of proteins calculated by the graph theory-based rigidity analysis of thermal unfolding simulation.	[105]
MD simulation	e.g., GROMACS	Analysis of protein unfolding pathways at higher temperatures.	[71]
B-Fitter	https://www.kofo.mpg.de/en/research/organic-synthesis, accessed on 23 April 2023	Calculates and averages the B-factor values for all atoms in an amino acid.	[71]
FoldUnfold	http://bioinfo.protres.ru/ogu/, accessed on 23 April 2023	Uses the expected average number of contacts per residue calculated from the amino acid sequence as an indicator for whether a given region is folded or unfolded.	[106]
PredyFlexy	https://www.dsimb.inserm.fr/dsimb_tools/predyflexy/, accessed on 23 April 2023	Combines the B-factor with the state of motion of amino acid residues during molecular dynamics simulations.	[104]
FIRST	--	Representation of protein structure as a set of constraints on bond-angle interactions, identification of rigid and flexible regions of protein conformation by CAN.	[107]
FlexPred	https://kiharalab.org/flexPred/, accessed on 23 April 2023	Flexibility in predicting elastic residues using the SVM algorithm.	[104]
Rosetta Design	Rosetta 3.13 software	Design of thermally stable proteins based on iterative sidechain optimization and backbone relaxation through optimizing packing and idealizing backbone conformation.	[100]
FRESCO	---	Combined with MD simulations to predict flexible regions of proteins that can incorporate stable disulfide bonds.	[108]
HINGEprot	http://bioinfo3d.cs.tau.ac.il/HingeProt/, accessed on 23 April 2023	Predicts the hinge region of a protein.	[104]
PROSS	http://pross.weizmann.ac.il, accessed on 23 April 2023	Calculation of ΔΔG and thus analysis of potential stable mutation locations using Rosetta combination sequences.	[109]
FireProtDB	https://loschmidt.chemi.muni.cz/fireprotdb/, accessed on 23 April 2023	Numerical data, structural information for mutation experiments with a variety of proteins.	[110]

**Table 6:** Protein engineering for enhancing the thermal stability of BGL.

Organism	Strategy	Mutations	Molecular Effects	Improved Characteristics	References
*Penicillium funiculosum* (PfBgl3A)	Rational design SDM	---	---	---	[113]
*A. tumefaciens 5A* (H0HC94)	Rational design SDM OEP	W127F, V176A, L178A, L178E	Enhancement of hydrophobic interactions	↑ T_1/2_~2 or 3-fold.	[87]
Metagenomic library of Turpan Depression (Bgl6)	Directed evolution Quikchange	V174C/A404V/ L441F	Enhancement of hydrophobic interactions within the enzyme	---	[85]
*Thermomicrobium roseum* (B9L147)	Rational design SDM OEP	V169C	--	↑ T_1/2_~2-fold.	[114]
*H. orenii*	Rational design SDM	V169C/E173L/ I246A	Increase in hydrophobic interactions	T_1/2_ > 7 h at 70 °C.	[84]
GenBank MK490918 (Bgl15)	Directed evolution Error-prone PCR Petri-dish-based double-layer high-throughput screening	S39T/L42N/ V167C/W178L/ A251L/E319A/ E326P/A396V/ L433F	Increasing hydrophobic interactions and formation of more additional hydrogen bonds	T_1/2_ from 0.8 h to 180 h at 50 °C.	[21]
*P. piceum H16*	Rational design Proline theory Computer-assisted virtual saturation mutation	S507F/Q512W/ S514W	Mutation of glycine by proline reducing conformational entropy Increased hydrophobic interactions	↑ By 46.3%.	[32]
*C. thermocellum* (BglA)	Directed evolution Error-prone PCR	A17S/K268N	Increasing hydrophobic interactions	↑ T_i_ by 6.4 °C.	[18]
GenBank FJ686869 (Bgl1D)	Directed evolution DNA shuffling	S28T/Y37H/ D44E/R91G/ L115N	Enhancing interaction with protein structure around water molecules and introduction of more hydrogen bonds	↑ T_1/2_~10-fold.	[24]
GenBank HV348683 (Ks5A7)	Directed evolution Error-prone PCR	T167I/V181F/ K186T/A187E/ A298G	Increasing hydrophobic interactions with the protein core	↑ T_1/2_~8640-fold.	[115]
*Coniophora puteana* (CpBgl)	Semi-rational design (HotSpot, Alanine scanning technique) SDM	Q20C, A240S	A combination of structural changes in the active pocket and protein–ligand interactions	↑ T_1/2_~5-fold.	[73]
MeBglD2	Rational design Directed evolution	His8/Asn59/ Gly295	Increasing hydrophobic interactions with the protein core	↑ T_m_ by 9 °C.	[116]

## Data Availability

Not applicable.

## Sample Availability

Not available.
